# Intrapulpal temperature changes during the cementation of ceramic veneers

**DOI:** 10.1038/s41598-022-17285-x

**Published:** 2022-07-28

**Authors:** Edina Lempel, Dóra Kincses, Donát Szebeni, Dóra Jordáki, Bálint Viktor Lovász, József Szalma

**Affiliations:** 1 Department of Restorative Dentistry and Periodontology, University of Pécs Medical School, Dischka Gy. Street 5, Pécs, 7621 Hungary; 2 Department of Oral and Maxillofacial Surgery, University of Pécs Medical School, Dischka Gy. Street 5, Pécs, 7621 Hungary

**Keywords:** Medical research, Materials science

## Abstract

Adhesive cementation of ceramic veneers may increase pulpal temperature (PT) due to the combined effect of heat generated by the curing unit and the exothermic reaction of the luting agent (LA). PT increase may induce pulpal damage. The aim was to determine the PT rise during the luting of ceramic veneers (CV) of different thicknesses with light- or dual-curing (LC, DC) adhesive cements as well as pre-heated restorative resin-based composites (PH-RBC). For this a thermocouple sensor was positioned in the pulp chamber of a prepared maxillary central incisor. LC, DC adhesive cements and PH-RBCs heated to 55 °C were used for the luting of CVs of 0.3, 0.5, 0.7, and 1.0 mm thicknesses. The exothermic reaction of LAs added significantly to the thermal effect of the curing unit. PT change ranged between 8.12 and 14.4 °C with the investigated combinations of LAs and ceramic thicknesses (p ≤ 0.01). The increase was inversely proportional to the increasing CV thicknesses. The highest rise (p ≤ 0.01) was seen with the polymerization of PH-RBCs. Temperature changes were predominantly influenced by the composition of the LA, which was followed by CV thickness.

## Introduction

Ceramic laminate veneers are widely used as esthetic restorations for the correction of unfavorable tooth appearance. The design of the preparation, tissue removal, as well as the thickness of the restoration is dependent on the specific indication for the ceramic veneer. Their proportionality is not unequivocal^1^. Although conventional tooth preparation for laminate veneers can be considered a reliable technique, minimally invasive or even ‘prepless’ veneers are regarded as additional methods applicable for tooth preparation^2^.

The clinical success and durability of a porcelain veneer is greatly influenced by the strength of the adhesion formed between the interfaces of the three different components; the porcelain veneer, luting agent and tooth surface^3^. A close apposition of the enamel and the porcelain surfaces has a synergistic effect on the overall bond strength of the enamel/luting agent/porcelain complex^4^. This complex is characterized by a higher bond strength compared to the sum of the individual attachments between the enamel/luting agent and luting agent/porcelain^4^. In order to provide this ultimate linkage, adhesive resin cements are typically used for the cementation of ceramic veneers. This also improves the fracture resistance of the inherently brittle ceramics^5^. Their good esthetic and mechanical properties as well as low solubility in the oral environment can further improve the quality of these restorations^3,6,7^. Within the group of adhesive cements, light-curing resin cements are generally preferred due to their longer working time and superior color stability^8^. Since the porcelain veneer absorbs between 40 and 50% of the emitted light, their thickness is the primary factor in determining light transmittance available for polymerization^9^. In case of a porcelain with a thickness of more than 0.7 mm, light-cured resin composites do not reach their maximum hardness^10^. In such cases, the use of dual-cured adhesive resin cements is advised. Failures associated with these adhesive resin cements are disintegration in marginal integrity and discoloration^1,11^.

The use of pre-heated restorative resin-based composites (RBC) as luting agents was first described in 1987^12^. Their application is enjoying increasing popularity in today’s clinical practice. Given their reduced viscosity, allowing for thin film thickness, good adaptation and the benefits of high filler content the use of pre-heated RBCs as luting agents has been extensively assessed by clinical studies and laboratory investigations^13–18^. The increased filler load improves mechanical- and bond strength, thereby improving fracture resistance^15,19^. In comparison to resin cements, pre-heated RBCs are also less expensive as well as offer a greater range of shades. Additionally, they decrease polymerization shrinkage and stress generation in the exposed cement layer at the bonded interface which imparts greater resistance to intraoral degradation^16^. Aside from these benefits however, pre-heated RBCs were also found to negatively influence the adaptation of fixed dental prostheses as reported by a recent systematic review^20^.

Regardless of the luting agent used, a high degree of conversion (DC) is key to achieving the previously described benefits through different thicknesses of ceramic veneers^21,22^. Although, the use of a high-irradiance curing unit or longer exposure duration can improve DC, these processes also involve thermal reactions which could potentially be hazardous to the dental pulp^23,24^. The temperature rise during the curing of resin-based dental materials is attributed to both the energy absorbed during irradiation with light-curing units and the exothermic reaction of polymerization^25–27^. Although, both the remaining dentin thickness and the ceramic veneer have temperature-shielding effects, the pulpal temperature (PT) rise may still exceed the presumed threshold of 5.5 °C, even without the use of a resin cement^28^. The exothermic reaction which characterises polymerization can further increase PT and is proportional with the amount of C=C bonds present in the given resin cement. The capacity of the inorganic compartments to absorb external energy also affect heat diffusion within the material^29,30^. According to a systematic review on porcelain veneers, the frequency of endodontic failure (1%) is similar to debonding (0.8%), however the overall clinical survival is still considered to be high^31^. An ex vivo study showed a temperature rise of 5.5 °C lasting for 40 s to lead to immediate cell damage^32^. The examined cells were protected against apoptosis by a complex interaction of molecular processes. Apoptosis was detected when temperatures reached 7.5 °C^32^. In support of this observation, in a recent in vivo study, the authors concluded that increased PTs may induce inflammatory reactions, even if the temperature rise does not exceed the previously defined threshold of 5.5 °C^33^. Whilst the real significance of pulpal temperature rise is still controversial, it is accepted that it should be kept as low as possible during dental procedures involving the polymerization of light cured materials^34,35^. The temperature increase detected during RBC polymerization is the sum of the exothermic reaction of polymerization and the energy absorbed during irradiation with light curing units (LCU)^25,26^. This topic has been investigated by several studies in the past. These have addressed in particular the impact of the radiant energy, spectral characteristics of the curing unit and the RBC type or shade as well as applied thickness on the temperature rise within the pulp chamber^36–39^. Most of the studies found the applied radiant energy to be the crucial factor in pulpal temperature rise. The exothermic reaction of the RBCs was also found to be a contributing factor^26,38–40^. The use of resin-based adhesive cements and pre-heated RBCs as luting agents for ceramic veneers are widely indicated since these materials can increase the fracture resistance of ceramic restorations. Adhesive luting is also a more conservative approach which preserves larger amounts of dental tissue^5,41,42^. Although, the thermal effect of LCUs and the exothermic polymerization of the adhesive luting material dominates during veneer cementation, the shielding effect of the ceramic veneer against light transmission may modify this unfavorable temperature rise within the pulp chamber. Taking light attenuation into consideration as a function of the restoration thickness, it is assumed that the temperature rise is strongly influenced by the interposing ceramic thickness^28,43^. Although data is already available on the thermal effect of resin-based materials as well as the shielding effect of ceramics, their combinatorial effect is less investigated. Therefore, the purpose of the present in vitro study was to compare the intrapulpal thermal changes resulting from the cementation of various thickness ceramic veneers with light- and dual-curing adhesive resin cements as well as pre-heated sculptable submicron and microhybrid bulk-fill RBCs. The null-hypotheses of the research were two-fold; (1) there is no difference in PT change using different luting agents during ceramic veneer cementation, and that (2) there is no significant influence of ceramic layer thickness on PT rise.

## Methods

### Resin-based luting agents, ceramic plates and radiant exposure

During this in vitro study the effects of four resin-based luting agents (RLA); a light-curing (LC) and a dual-curing (DC) adhesive resin cement, a pre-heated sculptable submicron-filled and a pre-heated sculptable microhybrid restorative RBC on PTs were analyzed. The brands, chemical compositions and manufacturers are presented in Table 1.

**Table 1:** Materials, manufacturers, classification and composition of the investigated adhesive resin cements and pre-heated resin-based composites.

Material (code)	Shade	Manufacturer	Classification	Resin system	Filler	Filler loading
Variolink Esthetic LC (VE_LC)	Light	Ivoclar Vivadent, Schaan, Liechtenstein	Light-curing adhesive resin cement	UDMA, 1,10-DDMA	0.04–0.2 μm ytterbium trifluoride and spheroid mixed oxide	38 vol% 64 wt%
Variolink Esthetic DC (VE_DC)	Light	Ivoclar Vivadent, Schaan, Liechtenstein	Dual-curing adhesive resin cement	UDMA, 1,10-DDMA	0.04–0.2 μm ytterbium trifluoride and spheroid mixed oxide	38 vol% 64 wt%
Estelite Sigma Quick (EQ_55 °C)	A1 E	Tokuyama Dental, Tokyo, Japan	Conventional submicron RBCpre-heated to 55 °C	BisGMA, TEGDMA	0.1–0.3 μm monodispersing spherical silica-zirconia filler; prepolymerized filler of silica-zirconia and copolymer	71 vol% 82 wt%
Filtek One Bulk Fill Restorative (FOB_55 °C)	A1	3M ESPE, St. Paul, MN, USA	Microhybrid bulk-fill RBC pre-heated to 55 °C	AFM, UDMA, AUDMA, 1,12-DDMA	20 nm silica, 4–11 nm zirconia, cluster Zr-silica, 0.1 µm ytterbium-trifluoride	58.5 vol% 76.5 wt%

*RBC* resin-based composite, *E* enamel, *BisGMA* bisphenol-A diglycidil ether dimethacrylate, *AFM* addition fragmentation monomer, *UDMA* urethane dimethacrylate, *AUDMA* aromatic urethane dimethacrylate, *1,10-DDMA* 1,10-dodecane dimethacrylate, *1,12-DDMA* 1,12-dodecane dimethacrylate, *LC* light-cure, *DC* dual-cure, *vol.%* volumetric , *wt%* weight %.

Medium translucent A1 shade lithium disilicate ceramic plates (7 × 7 mm) were fabricated from GC Initial LiSi veneering ceramic ingots (GC Initial LiSi Press; GC Europe, Leuven, Belgium) by heat-pressed method and were then fired and glazed from one side (veneered surface) according to the manufacturer’s instruction. The specimens were then finished to achieve an even and smooth surface using 220-, 400-, and 600-grit sandpaper under water-cooling. This was followed by polishing using silicone points. Ceramic plates, intended to represent the veneers, were fabricated in thicknesses of 0.3 mm, 0.5 mm, 0.7 mm and 1.0 mm. The final dimensions of each ceramic plate were confirmed using a digital caliper with 0.001 mm accuracy (ABS Digimatic Caliper; Mitutoyo, Tokyo, Japan). The ceramic specimens were not acid-etched or silanated and nor were they coated with an adhesive.

The single dose capsulated restorative RBCs were pre-heated to 55 °C in a composite warming device (Ena Heat; Micerium, Avegno, Italy) for 15 min. Each capsule was heated just once for the cementation of 1 specimen only. The resulting RBC temperatures were measured with a non-contact infrared (IR) optics coupled to a digital thermometer (TESTO 845; Testo Magyarország, Budapest, Hungary). The IR thermometer was able to register temperatures in an area as small as 1 mm^2^ (optical resolution of 75:1) with a resolution of 0.1/1 °C and a data sampling frequency of 10 measurements per second. For the measurements conducted during the actual luting with the pre-heated RBCs, the ceramic plates were also pre-warmed in the same device to reduce heat dissipation.

During the experiments the same Light Emitting Diode (LED) light curing unit (LCU) (LED.D; Woodpecker, Guilin, China; Λ = 420–480 nm; 8 mm exit diameter fiberglass light guide) was used operated in a standard mode for 40 s. This was powered by a line cord at room temperature of 24 °C ± 1 °C controlled by an air-conditioner. The positioning of the light guide tip was standardized ensuring each sample received the same light beam character. The radiant exitance (mW/cm^2^) and exposure (J/cm^2^) delivered by the light curing unit (LCU) were measured using a radiometer (checkMARC; Bluelight Analytics, Halifax, Canada). The radiant exitance at the tip of the LCU was measured by placing the tip at a standard distance of 1 mm from the radiometer sensor. The radiant exposure was also calculated. The light attenuation of the four different thicknesses of ceramic plates was measured similarly, by placing the tip of the LCU at a standard distance of 1 mm over the ceramic specimen which was positioned centrally on the sensor.

### Sample preparation for pulpal temperature measurements

All methods were performed in accordance with the Declaration of Helsinki principled. Ethical approval was granted by the Regional Research Ethical Committee of University of Pécs to use extracted teeth for research purposes (approval number: IRB: PTE/3795) and informed consent was obtained from the subject. The maxillary central incisor to be used in this study was removed for periodontal reasons (severe attachment loss indicating extraction). The freshly extracted, caries-free, cleaned tooth was initially kept in physiological saline. A single tooth model was chosen for all experimental trials to limit any effects from structural differences in enamel and dentin^26^. The vestibular surface was prepared and polished flat leaving a two-millimeter thin layer of enamel and dentin from the buccal wall of the pulp chamber. The enamel thickness to be removed was estimated and controlled by a digital intraoral radiograph. Five millimeters of the root apex were also removed to expose the root canal. All pulpal residues were removed with endodontic files. This was followed first by irrigation with 5.25% sodium-hypochlorite solution, then saline and finally dried with paper points. To allow the insertion of the 0.5 mm diameter Cu/CuNi K-type thermocouple probe (Type K thermocouple; TC Direct, Budapest, Hungary) a hole was created on the palatal cingulum of the tooth with a one-millimeter diameter cylindrical diamond bur. The thermocouple sensor was fixed to the dentin on the buccal wall of the pulp chamber by means of a thin layer of cyanoacrylate glue (Loctite Super Glue; Loctite, Düsseldorf, Germany). The palatal hole was closed with flowable RBC (Filtek Supreme Flowable; 3 M, St. Paul, MN, USA). The remaining enamel and dentin thickness as well as the position of the thermocouple were confirmed radiographically. The pulp chamber and root canal were subsequently injected with ECG gel (Aqua Sound Basic; Ultra-gel Hungary 2000, Budapest, Hungary) to replicate pulp tissue. Flowable RBC was used to close the apical orifice and the tooth was embedded in clear acrylic one millimeter below the cemento-enamel junction. The tooth was immersed in a water bath of 37.0 ± 0.5 °C. Temperature measurements and heat registrations were recorded by a digital thermometer (El-EnviroPad-TC; Lascar Electronics, Salisbury, UK) attached to the thermocouple, with resolution of 0.1 °C and frequency of one measurement per second.

**Figure 1:**
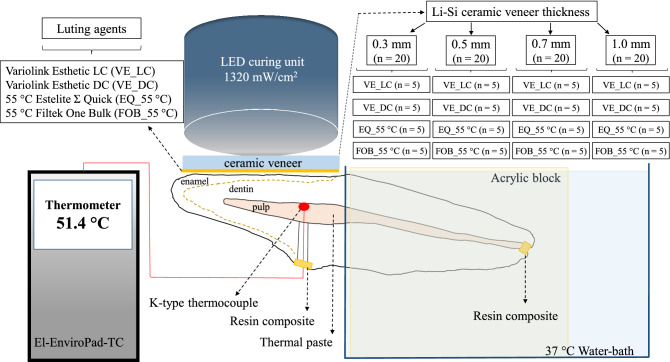
Schematic figure of the experimental set-up for pulpal temperature measurements.

Firstly, only the thermal effect of the LCU after a 40 s exposure was measured. To provide heat conduction, a transparent heat conducting silicone gel (8462 Silicone Grease; MG Chemicals, Burlington, Ontario, Canada; heat conductivity: 0.16 W/mK) was applied between the dentin and ceramic surfaces. The temperature changes during the cementation of 0.3 mm, 0.5 mm, 0.7 mm and 1.0 mm ceramic specimens, with the application of the four investigated RLA were measured (Fig. 1).

RLA of a standard volume was applied to the center of the non-glazed ceramic surface. This was then centrally oriented to the prepared tooth surface and manually loaded with a 5 N load in the case of resin adhesive cements, and 10 N load when pre-heated restorative RBCs were investigated. Measurements from preliminary tests showed that the manual placement of the ceramic body with a load of 5 or 10 N achieved a consistent RLA layer thickness of 100 ± 10 μm. An algometer was used to ensure the size of the load (Force Dial FDK 16; Wagner, Greenwich, USA). Excess luting agent was removed using a microbrush and the RLA was photoactivated through the ceramic plate for 40 s.

The cemented specimen was then swiftly removed and the thickness measured using the digital caliper. The film thickness was calculated from the difference between the thickness of the cemented specimen and the ceramic plate alone. There were a total of 16 groups for the various ceramic veneer/RLA combinations, and temperature measurements were recorded 5 times for each (n = 5).

Since no dental adhesive system or ceramic surface treatment was used, the polymerized RLA could easily be removed from both the tooth and ceramic plate following the measurements without leaving any deposits on either of the surfaces. This enabled the same tooth to be re-used for each measurement.

### Statistical analysis

Pilot study results and sample size formula were used to estimate sample size^44^.

Sample size formula: $n=\frac{{({z}_{1-\frac{\alpha }{2}}+{z}_{1-\beta })}^{2}{({s}_{1}+{s}_{2})}^{2}}{({M}_{1}-{{M}_{2})}^{2}}$

[z = standard score; α = probability of Type I error at 95% confidence level = 0.05; z_1−α/2_ = 1.96 for 95% confidence; β = probability of Type II error = 0.20; 1 − β = the power of the test = 0.80; z_1−β_ = value of standard normal variate corresponding to 0.80 value of power = 0.84; s_1_ = standard deviation of the outcome variable of group 1 = 0.27; s_2_ = standard deviation of the outcome variable of group 2 = 0.44; M_1_ = mean of the outcome variable of group 1; M_2_ = mean of the outcome variable of group 2; (M_1_—M_2_) = 0.5 if it is expected to detect 0.5 °C difference between two investigated groups as significant.] Using the formula N_final_ = $\frac{2n}{1-0.1}$ the predicted sample size (n) was found to be a total of 4.9 samples per group. According to the calculation n = 5 per group sample size was selected.

The statistical analyses were performed with SPSS (Version 26.0; IBM, Armonk, NY, USA). The normal distribution of the data was tested with the Kolmogorov–Smirnov test. The data was subsequently subjected to a parametric statistical test. The differences in light attenuation and temperature change were compared with one-way analysis of variance (ANOVA). Tukey’s post hoc adjustment was used for multiple comparison in all ANOVA models. Multivariate analysis (general linear model) and partial eta-squared statistics were used to test the influence and describe the relative effect size for luting *material* and ceramic *thickness* as independent factors. P values below 0.05 were considered statistically significant.

## Results

The remaining dentin-enamel thickness was 2 ± 0.2 mm based on the measurements taken at three different locations on the radiographic image of the tooth. The maximum radiant exitance of the LED LCU was 1320 ± 10 mW/cm^2^. The drop of total radiant exposure was significant (p < 0.01) through all the investigated ceramic thicknesses. There was a radiant exitance reduction of 30%, 40%, 50% and 60% through the 0.3 mm, 0.5 mm, 0.7 mm and 1.0 mm thick ceramic specimens, respectively. One-way ANOVA and Tukey’s post hoc tests indicated that the differences between the light irradiance transmitted through the ceramic plates of different thicknesses were statistically significant (p < 0.01).

**Figure 2:**
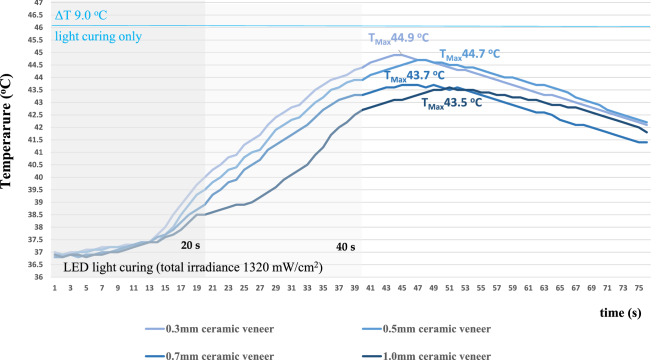
Representative registration curves of pulpal temperatures measured during irradiation through different thicknesses of ceramic veneers without resin luting agents.

In the case of PT changes, light curing without the interposition of ceramic veneers or RLA increased the 37 °C base temperature by 9 °C. The interposition of a ceramic plate (without RLA) limited this temperature increase and was found to be inversely proportional with the ceramic thickness (Fig. 2).

The statistical analysis did not find a statistically significant difference in light attenuation between either the 0.3 and 0.5 mm thick specimens, or the 0.7 and 1.0 mm thick ceramic veneers. Contrastingly, the difference was shown to be significant between 0.5 and 0.7 mm (p < 0.01). The 1.0 mm thick ceramic specimen reduced the thermal effect of the light curing unit by 2.5 °C, the 0.7 mm by 2.3 °C, the 0.5 mm ceramic by 1.3 °C and the 0.3 mm thick ceramic plate by 1.1 °C.

Overall, the highest temperature rise during the polymerization of different RLAs through different thicknesses of ceramic veneers was seen with the application of the pre-heated restorative RBC FOB_55 °C (p < 0.001), which was followed by the pre-heated restorative RBC EQ_55 °C (p < 0.001). With the above RLAs, a statistically significant difference in temperature increase was found when cured through the 0.3 mm (p < 0.001), 0.7 mm (p = 0.037) and 1.0 mm (p = 0.005) thick ceramic veneers. Generally, the pre-heated materials increased the pulpal temperature by approximately 3–3.5 °C which occurred precisely as the cement was applied to the tooth surface. The time between removal of the RBC from the heating device and application to the tooth surface took 3–4 s and additional 6–7 s were required to apply the veneer before light curing.

**Figure 3:**
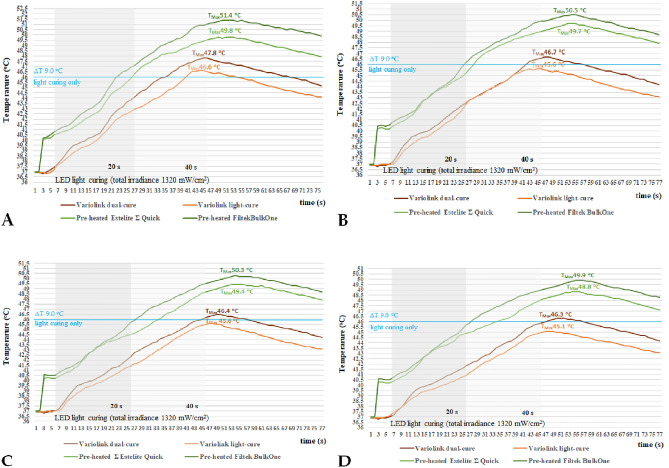
Representative registration curves of pulpal temperatures measured during irradiation of light-cured, dual-cured resin cements and pre-heated resin composites through 0.3 mm (**A**), 0.5 mm (**B**), 0.7 mm (**C**) and 1.0 mm (**D**) thick ceramic veneers.

When luting with the dual-curing adhesive cement (VE_DC) a lower temperature increase was found compared to the pre-heated restorative RBCs. The lowest temperature increase was recorded during the polymerization of the light-cure adhesive cement VE_LC (Fig. 3).

The results of the one-way ANOVA and Tukey's post hoc adjustment tests performed on the temperature recordings taken during the polymerization of ceramic veneers with different RLAs are presented in Table 2.

**Table 2:** Comparison of intrapulpal temperature change during polymerization of ceramic veneers with different luting materials.

Veneer thickness	Luting material	Mean ΔT (S.D.)	Comparison	Mean difference (°C)	95% CI		p-value
					Lower	Upper	
0.3 mm	VE_LC	9.56 (0.07)	VE_LC vs. VE_DC	− 1.36	− 2.06	− 0.66	< 0.001
			VE_LC vs. EQ_55 °C	− 3.38	− 4.08	− 2.68	< 0.001
	VE_DC	10.92 (0.15)	VE_LC vs. FOB_55 °C	− 4.84	− 5.54	− 4.14	< 0.001
	EQ_55 °C	12.94 (0.27)	VE_DC vs. EQ_55 °C	− 2.02	− 2.72	− 1.32	< 0.001
			VE_DC vs. FOB_55 °C	− 3.48	− 4.18	− 2.78	< 0.001
	FOB_55 °C	14.4 (0.27)	EQ_55 °C vs. FOB_55 °C	− 1.46	− 2.16	− 0.76	< 0.001
0.5 mm	VE_LC	8.58 (0.15)	VE_LC vs. VE_DC	− 1.24	− 2.11	− 0.36	0.005
			VE_LC vs. EQ_55 °C	− 4.18	− 5.05	− 3.31	< 0.001
	VE_DC	9.82 (0.15)	VE_LC vs. FOB_55 °C	− 4.92	− 5.81	− 4.06	< 0.001
	EQ_55 °C	12.76 (0.21)	VE_DC vs. EQ_55 °C	− 2.94	− 3.81	− 2.06	< 0.001
			VE_DC vs. FOB_55 °C	− 3.7	− 4.57	− 2.83	< 0.001
	FOB_55 °C	13.52 (0.26)	EQ_55^o^Cvs. FOB_55^o^C	− 0.76	− 1.63	− 0.11	0.1
0.7 mm	VE_LC	8.58 (0.18)	VE_LC vs. VE_DC	− 0.88	− 1.6	− 0.16	0.014
			VE_LC vs. EQ_55 °C	− 3.94	− 4.66	− 3.22	< 0.001
	VE_DC	9.46 (0.18)	VE_LC vs. FOB_55 °C	− 4.7	− 5.42	− 3.98	< 0.001
	EQ_55 °C	12.52 (0.27)	VE_DC vs. EQ_55 °C	− 3.06	− 3.78	− 2.34	< 0.001
			VE_DC vs. FOB_55 °C	− 3.82	− 4.54	− 3.09	< 0.001
	FOB_55 °C	13.28 (0.19)	EQ_55 °C vs. FOB_55 °C	− 0.76	− 1.48	− 0.04	0.037
1.0 mm	VE_LC	8.12 (0.18)	VE_LC vs. VE_DC	− 1.28	− 1.99	− 0.56	0.001
			VE_LC vs. EQ_55 °C	− 3.78	− 4.49	− 3.06	< 0.001
	VE_DC	9.4 (0.27)	VE_LC vs. FOB_55 °C	− 4.78	− 5.49	− 4.06	< 0.001
	EQ_55 °C	11.9 (0.11)	VE_DC vs. EQ_55 °C	− 2.5	− 3.22	− 1.78	< 0.001
			VE_DC vs. FOB_55 °C	− 3.5	− 4.22	− 2.78	< 0.001
	FOB_55 °C	12.9 (0.24)	EQ_55 °C vs. FOB_55 °C	− 1	− 1.72	− 0.28	0.005

One-way analysis of variance (ANOVA) and Tukey's post hoc adjustment.

*VE_LC* Variolink Esthetic LC, *VE_DC* Variolink Esthetic DC, *EQ_55 °C* pre-heated Estelite Sigma Quick, *FOB_55 °C* pre-heated Filtek one bulk restorative, *LC* light-cure, *DC* dual-cure, *S.D.* standard deviation, *CI* confidence interval.

**Figure 4:**
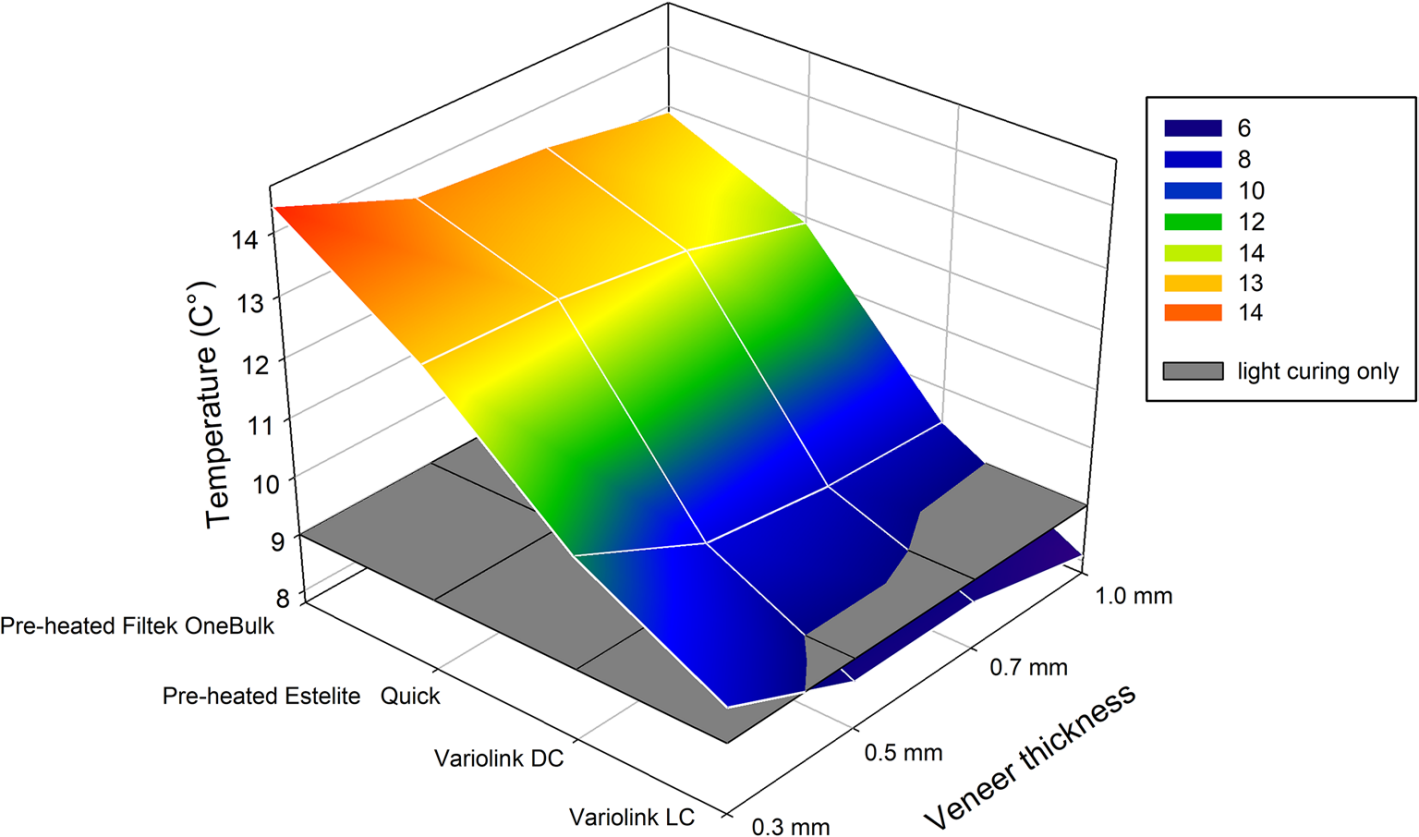
Pulpal temperature changes during polymerization of light-cured, dual-cured resin cements and pre-heated resin composites through different thicknesses of ceramic veneers. The grey layer shows the pulpal temperature rise caused by the light-curing unit alone. The image was constructed by SigmaPlot software (for Windows v. 12.0 build 12.2.0.45, Systat Software, Palo Alto, CA, USA).

As regards to the various ceramic veneer thicknesses, only the 0.3 mm thick specimen resulted in a significantly higher PT increase with the application of VE_LC, VE_DC, FOB_55 °C RLA (p < 0.001). On comparison of the 0.5 mm, 0.7 mm and 1.0 mm thicknesses, no significant rise in PT was demonstrated with VE_DC, EQ_55 °C and FOB_55 °C RLA. Statistically significant differences in temperature rise were measured between the 0.5 mm and 1.0 mm thick veneers (p < 0.002) and the 0.7 mm and 1.0 mm veneers (p < 0.002) with the application of VE_LC. The above-mentioned ceramic thicknesses in combination with VE_LC showed a heat dissipating effect on comparison to the thermal effect of the light-curing unit alone (Fig. 4). The results of the one-way ANOVA and Tukey's post hoc adjustment tests on the recorded intrapulpal temperature changes during the polymerization of various RLAs through different thicknesses of ceramic veneers are presented in Table 3.

**Table 3:** Comparison of intrapulpal temperature change during polymerization through different thickness of ceramic veneers.

Material	Veneer thickness	Mean ΔT (S.D.)	Comparison	Mean difference (°C)	95% CI		p-value
					Lower	Upper	
Variolink Esthetic LC	0.3 mm	9.56 (0.07)	0.3 vs. 0.5	0.98	0.69	1.27	< 0.001
			0.3 vs. 0.7	0.98	0.69	1.27	< 0.001
	0.5 mm	8.58 (0.15)	0.3 vs. 1.0	1.44	1.15	1.73	< 0.001
	0.7 mm	8.58 (0.18)	0.5 vs. 0.7	0	− 0.29	0.29	1
			0.5 vs. 1.0	0.46	0.17	0.75	0.002
	1.0 mm	8.12 (0.18)	0.7 vs. 1.0	0.46	0.17	0.75	0.002
Variolink Esthetic DC	0.3 mm	10.92 (0.15)	0.3 vs. 0.5	1.1	0.42	1.78	0.001
			0.3 vs. 0.7	1.46	0.78	2.14	< 0.001
	0.5 mm	9.82 (0.15)	0.3 vs. 1.0	1.52	0.84	2.19	< 0.001
	0.7 mm	9.46 (0.18)	0.5 vs. 0.7	0.36	0.32	1.04	0.45
			0.5 vs. 1.0	0.42	0.26	1.09	0.32
	1.0 mm	9.4 (0.27)	0.7 vs. 1.0	0.06	0.62	0.74	0.99
Pre-heated Estelite Sigma Quick	0.3 mm	12.94 (0.27)	0.3 vs. 0.5	0.18	− 0.95	1.31	0.97
			0.3 vs. 0.7	0.42	− 0.72	1.55	0.72
	0.5 mm	12.76 (0.21)	0.3 vs. 1.0	1.04	− 0.09	2.17	0.08
	0.7 mm	12.52 (0.27)	0.5 vs. 0.7	0.24	− 0.89	1.37	0.93
			0.5 vs. 1.0	0.86	− 0.27	1.99	0.17
	1.0 mm	11.9 (0.11)	0.7 vs. 1.0	0.62	− 0.51	1.75	0.42
Pre-heated Filtek One Bulk Restorative	0.3 mm	14.4 (0.27)	0.3 vs. 0.5	0.88	0.2	1.56	0.01
			0.3 vs. 0.7	1.12	0.44	1.79	0.001
	0.5 mm	13.52 (0.26)	0.3 vs. 1.0	1.5	0.82	2.18	< 0.001
	0.7 mm	13.28 (0.19)	0.5 vs. 0.7	0.24	0.44	0.92	0.75
			0.5 vs. 1.0	0.62	0.06	1.29	0.08
	1.0 mm	12.9 (0.24)	0.7 vs. 1.0	0.38	− 0.29	1.06	0.41

One-way analysis of variance (ANOVA) and Tukey's post hoc adjustment.

*S.D.* standard deviation, *ΔT* temperature change, *CI* confidence interval, *LC* light-cure, *DC* dual-cure.

**Figure 5:**
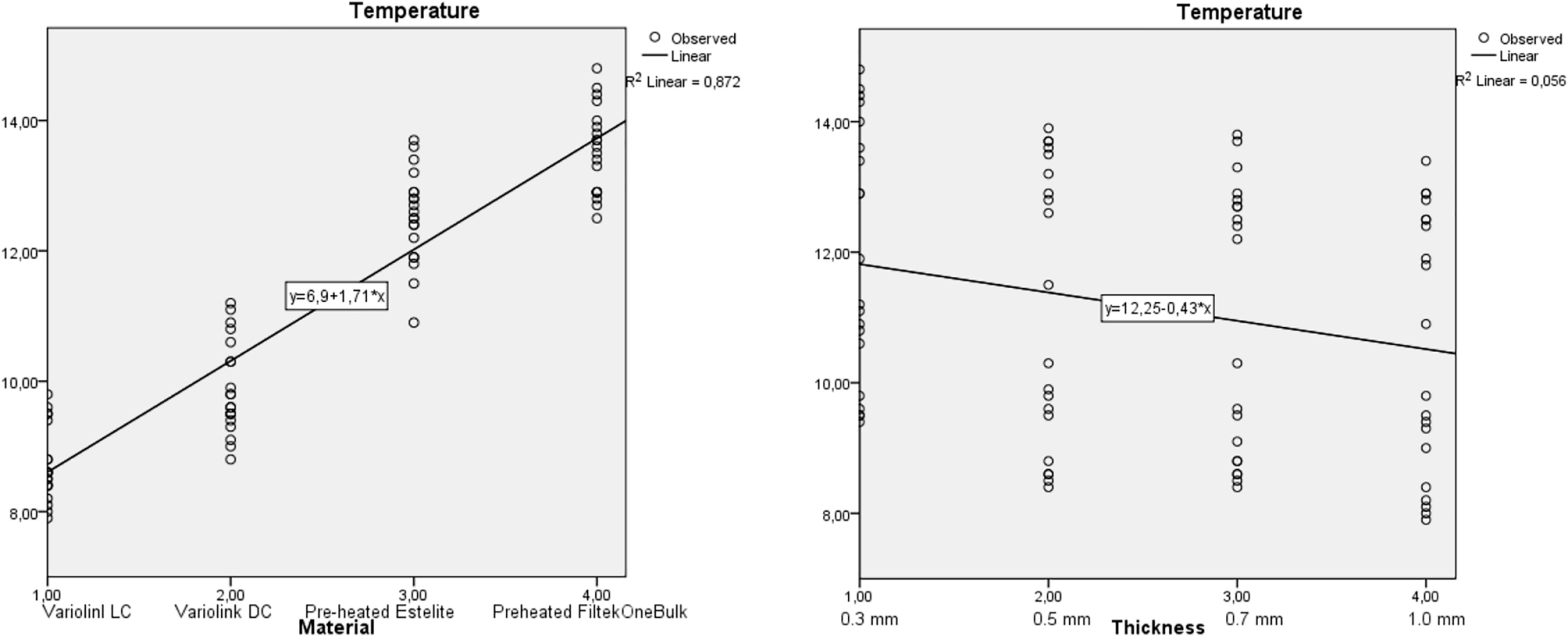
Effect of luting material and ceramic thickness on pulpal temperature change: (**a**) curve fit for material; (**b**) curve fit for thickness.

A 4 (*Material*) × 4 (*Temperature*) mixed-model ANOVA revealed that the main effect for both *Material* and *Thickness* on temperature change values were significant (p < 0.001) and the effect size was considered to be large (*Material* Partial ƞ^2^ was 0.96 and *Thickness* Partial ƞ^2^ was 0.64). The interaction of the two factors (*Material* × *Temperature*) did not show a significant effect (p = 0.28; Partial ƞ^2^ = 0.15) on the PT change during veneer cementation with the investigated materials. The high R^2^ value means a very close fit to the exponential regression lines (Fig. 5).

## Discussion

The present in vitro study demonstrated significant differences in PT changes when using light-cured, dual-cured adhesive cements, pre-heated restorative RBCs in combination with different ceramic thicknesses for veneer cementation. Hence both null-hypotheses were rejected. These results are consistent with other findings regarding the material-dependent temperature-increasing effect of RBC polymerization or the shielding effect of the interposed ceramic on PT rise^25,26,28,43^. General Linear Model demonstrated the importance of the RBC composition, as the effect size of the *Material* factor was found to be large on the PT rise. This is in line with a recent in vitro study where the effects of distinct types of RBCs on intrapulpal temperature rise were compared and the influence of the resin matrix system and filler content was found to be significant^26^. The effect size was shown to be medium for ceramic thickness implying that the shielding effect of the ceramic was less reliable compared to the exothermic effect of the adhesive luting agent. The present investigation highlights, that even though the interposed ceramic veneer attenuates the light intensity and thus the delivered energy for polymerization, the exothermic temperature rise associated with the adhesive luting agent may jeopardize pulp health.

In this study the measurements were carried out on a single permanent central incisor without the use of an adhesive system for all experimental groups to provide the same tooth-related conditions and eliminate any effect which may arise from structural and optical differences of the enamel and dentin. Although this provided a standard model to detect differences in temperature changes during the polymerization of RLAs, the lack of an adhesive layer application can be considered a limitation of the present study. The polymerized adhesive layer may serve as a barrier to heat transfer under clinical conditions. It is important to consider, that during polymerization of the adhesive layer the PT may increase faster than during the photocuring of the RBC, especially with an irradiance above 1000 mW/cm^2^^45^. Heat generated by the irradiation of the RBC could potentially compound thermal damage, as the pulpal and dentin temperatures are already higher due to the adhesive layer polymerization.

Thermal transfer to the pulp is strongly dependent on the thickness of the remaining tooth structure^28,46^. The thermal conductivity of enamel and dentin is ~ 0.45–0.93 W/mK and ~ 0.11–0.96 W/mK, respectively, which is considered to be low^47^. Increasing the thickness of the dentin results in an enhanced thermal-insulation effect^28^. One would assume that the increasing veneer thickness necessitates a proportional increase in the removal of tooth hard tissues, but in reality, veneer preparation design is strongly dependent on the specific indication. For instance, to align a buccoverted tooth, significant tissue removal could be required with a veneer of only minimal thickness. In contrast, for a palatoverted tooth, a thick veneer can optically align the tooth without any preparation. Consequently, the desired veneer thickness is not necessarily proportional to the amount of hard tissue removal. In the present study a standardized, conventional preparation design with ~ 2 mm remaining enamel / dentin thickness was employed for all the measurements in order to detect the effects of various veneer thicknesses and the type of the luting materials only. Although, the standard thickness of tooth structure could be a limitation of our study, considering the above-mentioned situations the 2 mm thick enamel/dentin in combination with different ceramic veneer thickness could be relevant clinical scenario.

Aside from the distance between the prepared tooth surface and the pulp, the effect of the pulpal blood circulation, perfusion rate, the volume and motion of the dentinal tubule fluid as well as the surrounding periodontal tissues also play important roles in heat conduction and protection against the rise of PT^48^. The clinical relevance of increased PT is the potential risk of thermal pulp damage^23^. Simulation of the pulpal circulation can decrease the intrapulpal temperature rise by two to four times compared to the results obtained in tests without simulation^49^. Although in vitro experiments demonstrated a material-dependent PT rise, which could exceed the 5.5 °C—considered to be the pathological threshold^26^—, it was concluded thereafter in an in vivo study, that neither a 2 mm deep cavity preparation nor the RBC polymerization resulted in a pulpal temperature rise approaching values that could represent a risk for thermal damage to the pulp^50^. As a limitation of our experiment, the study design lacked a simulation of the microcirculation. Although the tooth was held in a 37 ± 0.5 °C water bath for the duration of the study, this physiological temperature is not able to account for all the mechanisms by which heat is dissipated in vivo as the PT rises. The authors employed such a study design to illustrate the temperature changes which arise specifically due to the extent of the exothermic reaction which occurs in the investigated materials during veneer cementation. To provide the light required for the polymerization of the RLAs a second-generation LED LCU was used in this study with a radiant exitance of 1320 ± 10 mW/cm^2^ at a wavelength range of 420–480 nm. According to several previous experiments, the duration and intensity of the applied light seemed to be the most crucial factor in PT rise^24,35,51–53^. Results of the present study confirm this statement, since light-curing without the interposition of a ceramic veneer or RLA increased (ΔT = 9 °C) the PT by a significant degree. The extended exposure time (40 s) with a light irradiance of 1320 mW/cm^2^ resulted in 52.8 J/cm^2^ delivered radiant exposure. Because of the shielding effect of the ceramic and dentin during cementation, a high-intensity light or extended exposure time is recommended for complete polymerization. This also achieves acceptable mechanical properties and biocompatibility^21,54–56^. Whilst the increased delivered radiant exposure is beneficial to RLA polymerization, a strong positive correlation was found between energy density and PT rise^57^. Aside from the irradiance, the type and characteristics of the curing unit also have an impact on the amount of heat generated within the tooth^39,58^. During veneer cementation, ceramic interposition exhibits a temperature shielding effect. Results from the present investigation are consistent with the findings of previous studies which also showed an inverse relationship between ceramic thickness and temperature rise^28^. Our results showed, that the regression curve for temperature against the different thicknesses of ceramic showed a linear LCU energy loss with increasing thickness, which reflects the light attenuation occurring through an absorptive/scattering medium. Although the thickness of the ceramic veneer was found to be less significant in the pulpal temperature rise, as the difference proved to be significant between all the investigated veneer thicknesses only with the application of VE_LC. The linear regression model showed the value of the coefficient of determination to be only 5.6%. The shielding effect was lowest when polymerization occurred through the 0.3 mm thick ceramic veneers. The difference was significant compared to all thicker ceramics except when EQ_55 °C was used. Partial eta squared test showed that although the effect of ceramic thickness was significant on the pulpal thermal change the size of its effect was less when compared to that of the material’s. Regarding the relationship between resin cement hardness—which may reflect the degree of conversion—and ceramic type, thickness, translucency, and curing time, it was demonstrated that the effect size of ceramic thickness is the lowest among the other factors^59^. Aside from the light and heat attenuation capacity of ceramics, the shielding effect of increasing thickness of dentin may be more pronounced^28^.

Incomplete polymerization of the adhesive cement can lead to decreased bond strength, release of potentially toxic residual monomers, color change, increased risk of microleakage and debonding^21,54,60^. The heat energy released by the intense or extended lighting however, could cause injury to vital pulp tissues depending on the extent and length of the heat exposure^32^. Besides the characteristics of the employed light, the exothermic polymerization reaction of the luting agent also plays a significant role in PT rise. Our study design together with the single tooth model^25,26^ ensured uniform conditions allowing the comparison of the exothermic thermal change between different RLAs only. Previous researches found the exothermic reaction to be proportional to the amount of resin matrix available for polymerization and also found that the inorganic compartments affect heat diffusion within the material by their capacity to absorb external and internal energy^26,29,30^.

Furthermore, the type and concentration of the photoinitiator/accelerator system and the polymerization conditions also have an impact on the thermal change of the RBC during polymerization^61^. The latter includes, among others, the layer thickness of the applied material, spectral characteristics of the LCU, exposure time and the pre-polymerization temperature of the RBC^62–64^. Since the experimental conditions were standardized, the differences in the thermal change of the RLAs can be attributed to the different components. Regarding the composition of the investigated materials, although the light- and dual-cure RLAs have the same resin matrix/filler ratio, the dual-cure showed a significantly higher temperature rise compared to the light-cure RLA. The interpositioning of a ceramic veneer can reduce not only the degree of conversion in the case of the light-cure but also the dual-cure resin cement^65,66^. In the latter, the chemical setting allows for a more efficient polymerization compared to the light-cured resin cement^21^. Dual-cured resin cements are therefore able to compensate for decreased light transmission and may convert monomers to polymers more efficiently even with increased ceramic thicknesses. Since however, the polymerization of dual-cure RLA progresses to a higher degree, the accompanying exothermic reaction can further increase the PT as it was demonstrated also by our results.

Pre-heated restorative RBCs are used increasingly as luting agents for ceramic restorations due to their advantageous properties^14,67^. Although pre-heated RBCs have demonstrated better color stability, marginal adaptation, degree of conversion and strengthen ceramics^13,68,69^, the results of the present study warn of their potential hazard to pulp health as a result of their stronger thermal effect. Increased pre-cure temperature of the pre-heated RBCs elevated the PT by approximately 3 °C immediately, at the moment of the veneer placement. The temperature continued to rise with photocuring as the result of the heat generated by the light exposure and exothermic reaction. This may increase the degree of conversion and crosslinking by enhancing free radical and monomer mobility and intensifying collisions among molecules^70,71^. According to our results, the highest temperature rise was recorded with the use of FOB_55 °C followed by that of EQ_55 °C. On comparing the filler-matrix ratio, FOB contains 58.5 vol% inorganic component while EQ has 71 vol% filler content.

It means, that the former’s monomer ratio, thus the amount of C=C bonds is higher, leading to more a pronounced exothermic reaction. Furthermore, the chemical composition of the monomer matrix also has a strong influence on the polymerization kinetics. EQ is a BisGMA/TEGDMA based RBC, while FOB’s matrix is composed of UDMA, AUDMA, 1,12-DDMA, AFM monomers. BisGMA is considered to be the most viscous molecule among the above-mentioned monomers due to the strong intramolecular hydrogen bonding. This results in a limited rotational freedom, thus leading to reduced mobility and reactivity of this monomer during the polymerization process^72^. On the other hand, UDMA represents a combination of relatively high molecular weight with a high concentration of double bonds and low viscosity. This was shown to reach a higher degree of conversion and is characterized by an enhanced exothermic reaction^73^. Additionally, it is reasonable to assume a role also for a further FOB constituent, the so-called AFM, an addition-fragmentation chain transfer dimethacrylate monomer. This molecule participates readily in network formation by copolymerizing with multifunctional methacrylates further increasing the release of exothermic heat^74^. In addition to the monomer system, the filler-matrix ratio is also decisive regarding the degree of monomer to polymer conversion and exothermic heat release. EQ filler loading is higher compared to the filler content of FOB, which may restrict light penetration and the mobility of monomers and radicals.

It should be noted, that even though the partial eta-squared statistics demonstrated a high impact for the type and composition of luting material on pulpal thermal change, the results cannot be directly extrapolated to other RLAs.

Despite the limitations of this in vitro study, it can be concluded, that the intrapulpal temperature can increase above 8.12 °C during veneer cementation, using different combinations of RLA materials and ceramic thicknesses. The temperature values were predominantly influenced by the composition of the RLA materials followed by the thickness of the ceramic veneer. Moreover, the application of pre-heated restorative RBCs as luting agents resulted in a significantly higher temperature rise compared to that measured in the case of adhesive resin cements.

## Data Availability

The datasets generated during and/or analyzed during the current study are available from the corresponding author on reasonable request.
